# The Main Risk Factors of Nipah Disease and Its Risk Analysis in China

**DOI:** 10.3390/v10100572

**Published:** 2018-10-19

**Authors:** Jiarong Yu, Xinbo Lv, Zijun Yang, Shengbin Gao, Changming Li, Yumei Cai, Jinming Li

**Affiliations:** 1 Shandong Provincial Key Laboratory of Animal Biotechnology and Disease Control and Prevention, Shandong Agricultural University, 61 Daizong Street, Taian 271018, China; yujiarong19930921@163.com (J.Y.); xinbo-1122@163.com (X.L.); 15165853927@163.com (Z.Y.); gaoshengbin930106@163.com (S.G.); lcm614678030@gmail.com (C.L.); 2 China Center for Animal Health and Epidemiology, Qingdao 266000, China

**Keywords:** Nipah virus, fruit bat, transmission route, risk analysis

## Abstract

Nipah disease is a highly fatal zoonosis which is caused by the Nipah virus. The Nipah virus is a BSL-4 virus with fruit bats being its natural host. It is mainly prevalent in Southeast Asia. The virus was first discovered in 1997 in Negeri Sembilan, Malaysia. Currently, it is mainly harmful to pigs and humans with a high mortality rate. This study describes the route of transmission of the Nipah virus in different countries and analyzes the possibility of the primary disease being in China and the method of its transmission to China. The risk factors are analyzed for different susceptible populations to Nipah disease. The aim is to improve people’s risk awareness and prevention and control of the disease and reduce its risk of occurring and spreading in China.

## 1. Introduction

### 1.1. Virus Characteristics

The Nipah virus (NiV) belongs to the paramyxovirus family and the Henipavirus genus. In addition to thia, the Henipavirus genus also has four virus members: the Cedar virus (CedV), Kumasi virus (KV), Mòjiāng virus (MojV) and Hendra virus (HeV) [1,2]. Due to its ease of transmission and high lethality, the World Organisation for Animal Health (OIE) lists Nipah as a Biosafety Protection Level 4 (BSL-4), and the US Center for Disease Control and Prevention (CDC), and the National Institute of Allergy and Infectious Diseases (NIAID) list it as a Class C priority pathogen [3].

Henipaviruses are lipid-encapsulated viruses that form spherical to polymorphic, sometimes filamentous, particles. Wrapped in the virus particles is the filamentous nucleocapsid, which is made of viral RNA and nucleoproteins, and is associated with the RNA-dependent RNA polymerase and phosphoprotein [4] The diameter of an entangled filamentous nucleocapsids is about 1.9 μm, and the length of the envelope membrane about 17 nm [5]. The NiV genome has six structural genes. From the 3 ‘end, N proteins (nuclear proteins), P proteins (phospho-proteins), M proteins (matrix proteins), F proteins (fusion proteins), G proteins (glycoproteins) and L proteins (large proteins) are coded sequentially, and between each two genes, there is a non-coding region [6]. Among them, phospho-proteins are the largest proteins and are encoded by the P gene of HeV and NiV. In addition to phospho-proteins, the P gene also encodes the V and C proteins. The P gene of HeV also has a small ORF (the open reading frame) located between the coding regions of the C and V proteins which is used to encode basic proteins such as the SB protein (a short basic protein). However, this ORF is not found in NiV [7,8]. The main cellular targets of NiV are microvascular endothelial cells, epithelial cells, and neurons, and fusion proteins (F) and glycoproteins (G) can mediate syncytia formation [9,10]. Since N proteins are highly immunogenic, they can stimulate the body to produce antibodies at an early stage, and their antibody titer is high and has a long duration, so they can be used as a detection antigen for NiV [11]. NiV and HeV are rapidly inactivated in dry environments and both viruses survive for less than 15 min at 37 °C [12]. The Nipah virus is an enveloped virus; the virus is easily destroyed by soaps and detergents [13].

### 1.2. Epidemic Situation

NiV was first discovered in the state of Negeri Sembilan in Malaysia in 1997 [14] and was subsequently identified as a new virus that seriously harms the health of domestic pigs and humans. From September 1998 to April 1999, large-scale outbreaks of acute respiratory syndrome in pig herds occurred in Malaysia, causing 265 pig workers to become infected, 105 deaths, a fatality rate of about 40%, and 1.16 million pigs being culled [15]. Due to pig trading between Malaysia and Singapore, there was also a report of an NiV outbreak in Singapore in March 1999, which resulted in 11 people being infected and one death [16]. Since then, India, Siliguri, Kampuchea, Thailand, and other nine countries have reported the disease [17,18]. In Australia, although there has been no reports of the Nipah virus, the Hendra virus which is similar to the Nipah virus was first reported in Queensland, Australia in 1994 [19]. That incident caused 20 horses to become infected and one human fatality. Between 1994 and 2014, there were nearly 50 reports of outbreaks of this virus in Australia. All of the infected animals were horses.

**Figure 1:**
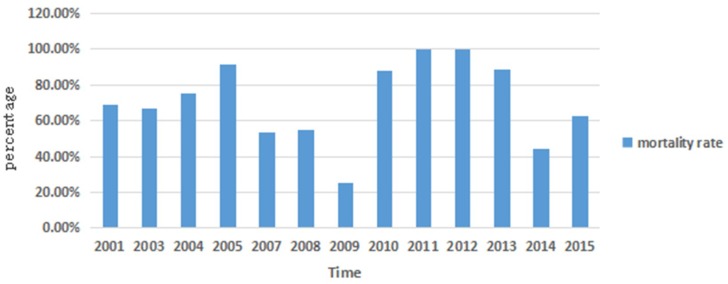
Death rate from Nipah virus (NiV) outbreaks per year in Bangladesh.

From 2001 to 2015, there were reports of outbreaks of NiV in Bangladesh in almost every year (Figure 1). There were a number of cases of Nipah virus infection in Bangladesh (mainly in the Western and Northwestern parts), with 249 reported infection cases and 176 deaths—a death rate of about 71%. In 2005, of 12 cases, 11 died, and the mortality rate was 91.8%. In 2011 and 2012, all 30 cases resulted in death—a mortality rate of 100% [20].

### 1.3. Nipah Disease-Bearing Animals and Major Incidence Populations

**Figure 2:**
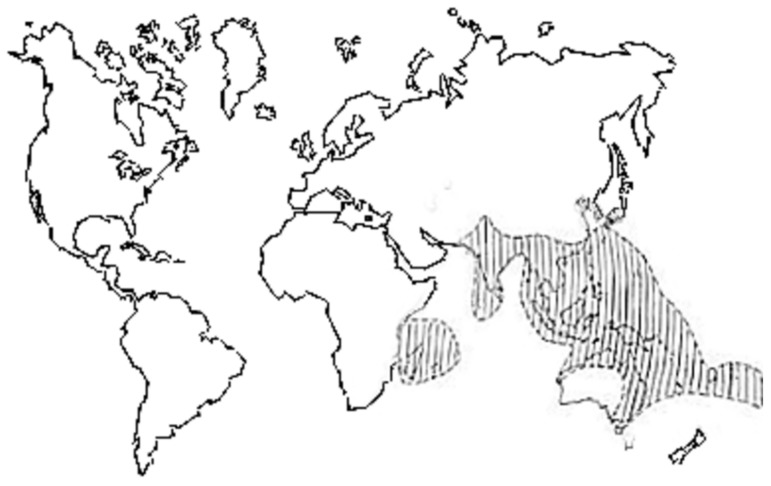
World distribution of fruit bats [30].

Current research indicates that fruit bats of the Pteropus genus are the natural hosts of NiV [21]. They are mainly distributed in South Asia, Southeast Asia, and Australia (Figure 2) [17]. They perch on trees [22] and feed on fruits, flowers, and pollen. In Australia, researchers conducted radiographic tracking studies showing that fruit bats, or flying foxes, have a flying range of up to 600 km [23]. Ephrin-B2 and -B3 were identified as the host cellular receptors for henipaviruses [24,25]. This also causes NiV to infect more animals, including pigs, humans, dogs, horses, goats, cats, and rodents. It has been reported that even cows are infected [26]. Among these susceptible animals, pigs and humans are the most susceptible animals.

#### 1.3.1. Infections of NiV among Pigs

In the first outbreak of NiV in Malaysia in 1998, pigs played a very important role as intermediate hosts [27]. The pigs infected in that incident mainly ate the scattered fruits that were partially eaten by the fruit bats. NiV has a 100% infection rate in pigs, but the mortality rate of infected pigs is not very high [28].

#### 1.3.2. The Outbreaks of NiV in Human

Humans are infected with NiV mainly through contact with the secretions and body fluids of infected pigs and humans, or by corpses. In the epidemic of NiV in Malaysia, the diseased population was mainly infected with NiV after exposure to secretions of sick pigs [27]. Among the 265 infected humans, 105 (39%) died [14]. Human-to-human transmission has been described during outbreaks in Bangladesh, where humans were infected with NiV by touching a patient’s saliva, body fluids, or corpse [29].

### 1.4. Clinical Characteristics and Harm of Different Susceptible Animals Infected with Nipah Disease

As the main disease populations of Nipah disease, the clinical symptoms of pigs and humans are the most typical. Pigs infected with NiV mainly show acute febrile disease, including dyspnea, increased salivation, and serous or bloody pus in the nasal cavity, in addition to neurological symptoms such as delirium and epilepsy, nystagmus, and obvious paralysis symptoms. In humans, the main organs affected by the Nipah virus are the brain and lungs [31]. Patients infected with the NiV-Malaysia strain develop clinical symptoms similar to encephalitis [28,32] and those infected with the NiV-Bangladesh strain develop respiratory diseases as well [29]. Due to the high infectivity and high lethal nature of NiV, there is a lot of information about the mortality rate of the reporter. To date, up to 600 cases of NiV infection have been reported, with mortality rates of up to 100% in some cases [33]. In Bangladesh, the mortality rate of infected people between 2001 and 2015 was as high as 75% [34]. In 2012, the death rate in the Joypurhat area of Bangladesh reached 100% [35].

## 2. The Route of Transmission of Nipah Disease in Different Epidemic Countries

The HeV-infected hamster model indicates that the virus is transmitted by direct contact, rather than by aerosol [36]. The zoonotic transmission efficiency observed during the outbreaks of the NiV-Malaysia strains and the HeV can be explained by the differences in initial replication sites between the HeV and NiV-Malaysia strains [37]. A ferret model test can better explain the spread of NiV in Bangladesh. The results of the model showed that the concentration of NiV-Bangladesh in the oral secretions is higher than that of NiV-Malaysia [38]. This explains why there are more reports of the spread of NiV between humans in Bangladesh.

### 2.1. The Fruit Bat-to-Human and Human-to-Human Routes

In Bangladesh, the most common route of infection is through the consumption of raw date palm sap contaminated with fruit bat urine or saliva, especially in Western Bangladesh [16,39]. In local customs, people have the habit of collecting fresh date palm juice to make tari. The earthen jars are recycled by local residents without being cleaned. Those that have been eaten by fruit bats contaminate the earthen jars and pollute the next tari, which infects the next purchaser of tari [40]. According to the statistics of the related literature, although there were many reasons for NiV infection in Bangladesh between 2001 and 2012, more than half of cases were caused by drinking fresh date palm juice [41]. Nipah virus was first isolated from patients with similar encephalitis in 1999. Among these patients, some had respiratory diseases [42,43]. In 2001, India reported that NiV can be transmitted to patients’ families and medical staff through the saliva and droplets of patients [44]. In Bangladesh, family members and medical staff were also infected because doctors do not wear gloves and masks when giving patients a check and the patient’s family do not take appropriate protective measures when they visit. In addition, the possibility of infection is highly increased while taking care of NiV patients [20,45]. In addition, in accordance with the local customs in Bangladesh, the relatives of the patients have a ceremony that involves kissing the deceased and cleaning the body fluids of the deceased during the burial. However, they usually do not wash their hands after the ceremony. So, this kind of contact without any protective measures also increases the risk of infection [29].

### 2.2. The Route of Fruit Bats to Livestock and Then to Humans

Livestock feeding on wild fruits contaminated by bats with NiV can be infected with the virus, and the infected animals further spread the virus to other animals, including humans. Pigs are infected with NiV after swallowing fruit buds or saliva-contaminated fruits. In 2014, the Philippines reported an NiV incident. The outbreak in the Philippines was likely caused by the Nipah virus. In this incident, 17 people were infected, nine died, and the death rate was 53% [46]. Although the route of NiV infection of the horses was unclear, fruit bats were detected in the outbreak area. It is speculated that the infection was caused by the horses eating the fodder contaminated by the urine and the feces of fruit bats [47].

### 2.3. The Route of Fruit Bats to Humans

There are many types of bats with 188 species from 42 genera. In addition, there are 59 species of fruit bat in the Pteropodidae [30,48]. Fruit bats are distributed mainly in South Asia, Southeast Asia, Australia, and Africa. In the Cameroon region of Africa, a virus similar to Henipavirus was detected from the serum of some local people. It is understood that these infected people were mainly those engaged in the slaughter of local bats. Due to the cross-reactivity of serum with NiV and HeV, a virus closely related to the henipaviruses appears to circulate in this region [49]. In Cambodia, although Nipah-positive antibodies were not detected in humans, antibodies to an NiV-like virus were detected in the body of Pteropus lylei [18]. In addition, local people are also engaged in bat slaughter work, which also carries the risk of NiV infection [50].

**Figure 3:**
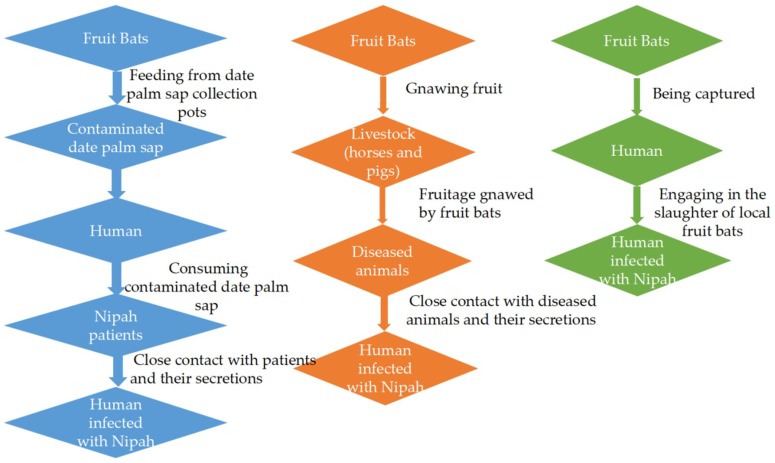
NiV’s transmission pathways in different countries. Blue: transmission routes in Bangladesh; orange: transmission routes in Malaysia and the Philippines; green: transmission routes in Africa and Cambodia.

By using Figure 3, we can further understand the NiV propagation mode in different countries.

## 3. Risk Analysis for the Possibility of Developing Nipah Disease in China

In Hainan, Guangxi and other places in Southwestern China, there are fruit bats which are the natural host of the Nipah virus. In these areas, pigs are mainly raised free range, which presents the risk of human infection with the Nipah virus through pigs infected by fruit bats. Moreover, China imports a large number of pigs from abroad every year, and there is also the risk of Nipah disease being introduced into China. Because of the ease of spread and high lethality of Nipah disease, it is necessary to conduct a risk analysis on the outbreak of Nipah disease in China.

### 3.1. Risk Analysis for the Possibility of Outbreak of NiV in China

With the continuous development of China’s economy, China has traded with foreign countries more frequently and inevitably trades with the Nipah disease countries. According to reports, among the neighboring countries bordering China, Nipah disease has also occurred in Siliguri, India [44]. Although Thailand and Cambodia have not reported Nipah disease, they have also confirmed the presence of NiV antibodies in their domestic fruit bats [50,51], indicating that there may be a risk of Nipah virus being introduced into China when they traded in the border areas of China with Nipah or a potential Nipah countries. This article analyzes the risk of Nipah disease being introduced to China.

#### 3.1.1. Live Pig Trade

Pigs are important intermediate hosts in the outbreaks of NiV in Malaysia [27]. The outbreaks of Nipah disease in Singapore are due to live pig trade with Malaysia [23]. If NiV is introduced to China, the first risk factor to consider is the pig trade. As the number one country for breeding pigs in the world, China needs to introduce a large number of breeding pigs from foreign countries every year [52]. According to the data, in 2017, among the six introduced countries—the United States, Canada, Denmark, the United Kingdom, France and the Netherlands—the United States, Canada, and France became the three major exporters of pig breeds to China [53]. Although there is no trade in breeding pigs between China and Southeast Asian countries, this does not mean that pigs from Southeast Asian countries will not enter China. The data show that the incubation period for pigs infected with NiV is from 4 days to 2 months [29]. By smuggling, pigs from countries such as Vietnam and Cambodia adjacent to China could enter China [54]. Once pigs carrying Nipah virus in smuggled pigs are raised with local rural pigs on the border, they may cause infection in the herd and even further infect the breeder causing outbreaks of Nipah disease.

#### 3.1.2. Personnel Flow

**Figure 4:**
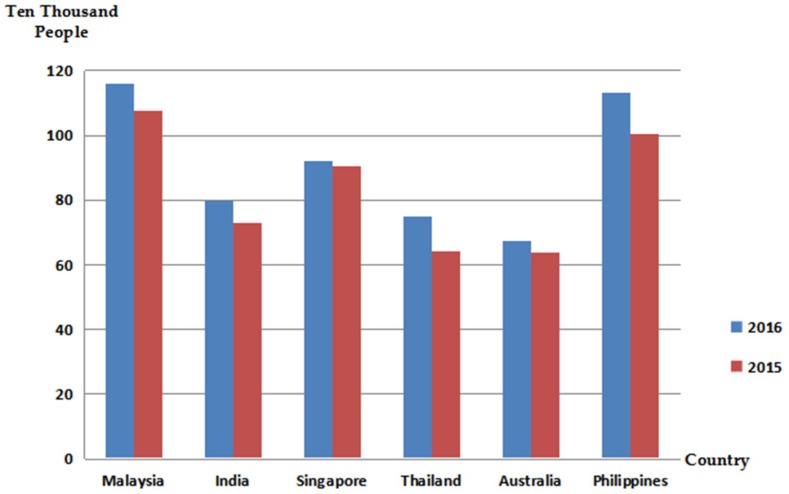
Statistics on the number of passengers coming to China from Southeast Asia and Australia. Note: the data comes from the Civil Aviation Administration.

With the improvement of the economic level and the increasing convenience of transportation, the number of tourists from Southeast and South Asia to China has gradually increased. Figure 4 shows the number of people who traveled to China from Southeast Asian in recent years. The incubation period for human infection with NiV is 6–11 days [55], and there are no clinical symptoms during the incubation period. The clinical symptoms of Nipah infection are similar to those of encephalitis, for example, fever, headache, cough, and decreased consciousness [28,32], which make it difficult to judge according to clinical symptoms. This poses the risk of Nipah virus being introduced into China through members of an epidemic country. There could be an increased risk of NiV transmission in China once introduced due to modern means of transportation. In addition, there have also been cases of Nipah virus infection in dogs and cats [20]. If these infected pets follow their hosts to China, the virus will also be introduced into China. Moreover, due to a lack of reports on the Nipah virus in China, the inspection and quarantine procedures for the virus are not perfect, and the potential risk of its introduction into China through pets still exists.

### 3.2. Risk Analysis of the Origin of Nipah Disease in China

**Figure 5:**
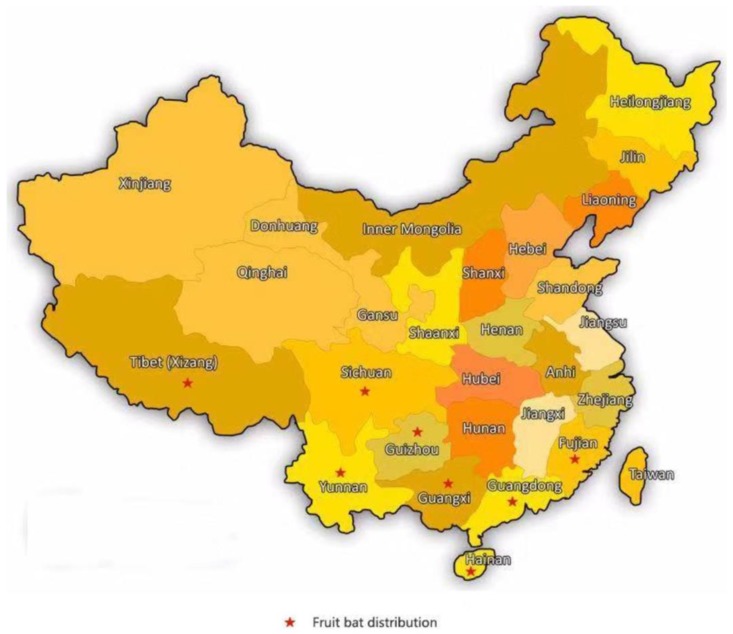
Distribution area of Chinese fruit bats.

At present, some institutions in China have initially established real-time quantitative RT-PCR methods and indirect ELISA methods for the detection of Nipah virus, which are used to prevent the introduction of Nipah disease, but they ignore the possibility of China being a source of the disease. As shown in Figure 5, fruit bats are mainly distributed in South China, including Hainan, Fujian, Guangdong, Guangxi, Yunnan, and Tibet [30,56]. This shows that China is at risk of having active Nipah disease.

#### 3.2.1. Fruit Bats

The natural host of the NiV is fruit bats of the genus Pteropus (“Flying bat”) [57]. Fruit bats are the main risk factor for the development of Nipah disease in China. During seasonal migration, many species of bats fly long distances [58]. According to reports, fruit bats can travel from Pakistan to Australia across South and Southeast Asia along the coast of Southern China [48]. This long-distance flight capability of fruit bats plays an important role in the spread of the virus. This indicates that in areas where the fruit bats or flying fox pass, susceptible animals can get Nipah disease from the pomace fed on by the fruit bats, and then, the infected animals are exposed to healthy animals or other susceptible groups, causing the virus to spread between groups, which causes the spread of Nipah disease. Therefore, research on the areas of fruit bats, favorite fruits, and other living characteristics can allow better analysis of the possibility of Nipah disease originating in China.

#### 3.2.2. Live Pigs

**Figure 6:**
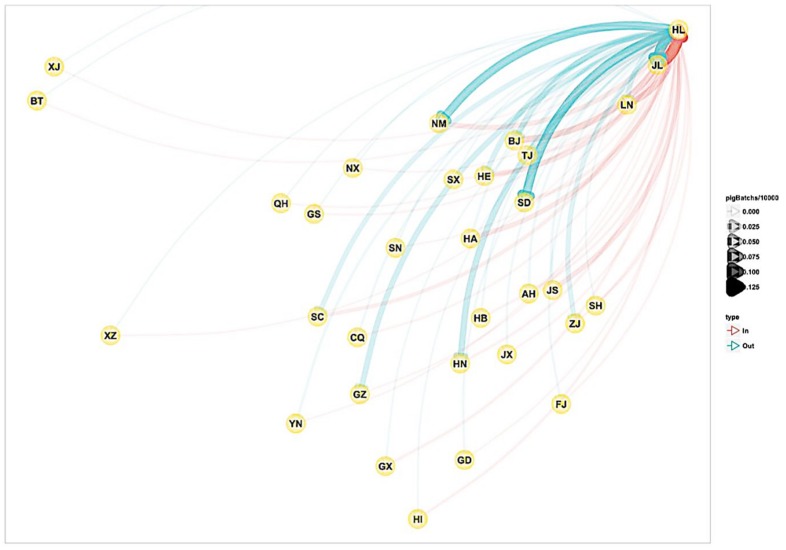
Live pig operation map (the letters in the figure represent abbreviations of China provinces) provided by the China Center for Animal Health and Epidemiology.

Due to the increase in large-scale breeding of pigs in China and the promulgation of the ecological pig raising policy in recent years, the small- and medium-sized pig farms have been strongly affected. According to the China Animal Husbandry Yearbook statistics, during the period 2008–2014, the number of pig farmers in the country decreased by 22.845 million in six years. However, the small and medium-sized pig farms have not disappeared as yet. The owners of small-scale farms who have sold less than 500 pigs in a year still account for 99.6%, and the number of slaughtered pigs accounts for 58.2% of the total number of 735 million heads in the country [59]. This indicates that the free-range breeding model of pigs in China still dominates. The pigs in the distribution area of fruit bats have a high chance of eating residues and seeds when they are foraging, and the risk of becoming infected with NiV is high. Now that China’s free-range pig industry employers are mostly farmers, their cultural level is low, and they lack the knowledge of scientific farming techniques and prevention and control measures [60]. Once a pig is infected with NiV, pig–pig and pig–human transmission develops. Although Yunnan (YN), Guangxi (GX) and other places are not large pig operation provinces, they also participate in domestic pig trade (Figure 6). If a sick pig carrying NiV enters the domestic pig trade network, NiV will spread rapidly throughout the country through transportation, and it cannot be controlled.

#### 3.2.3. Fruit Trade

China’s Guangxi, Hainan, Yunnan, and other regions are rich in longan, lychee, banana, mango, pineapple, and other fruits and wild fruits—foods eaten by fruit bats. Brown fruit bats also like to suck sugar cane, kapok, nectar, and other sugar-rich fruits in winter [61]. Every year, China has such fruits sold from Hainan and other places. Studies have shown that the virus can survive in juice or pulp, and at 22 °C, NiV can remain alive in the date palm juice for 7 days [39]. In mango meat, the virus can be stored at 22 °C or 37 °C for 48 h, and in lychee and papaya juice, it can be stored at 22 °C or 37 °C for 96 h [12]. The above situations indicate that the Nipah virus has a certain stability in pulp or juice. In Bangladesh, there have been cases of NiV infection caused by drinking date palm juice contaminated with fruit bats [40], and now, the convenience of transportation allows fruits to reach consumers in a short time period. If the fruits carry the Nipah virus, people will be infected with NiV if they do not peel and wash the surface of the fruit before eating.

#### 3.2.4. Fruit Farmers

In Bangladesh, there have been cases of infection with NiV due to drinking fresh date palm sap [62]. In the area where fruit bats are distributed in Southwestern China, fruit growers who plant longan and lychee are also at risk of contracting NiV. If you do not wash your hands with soap in time when collecting and handling the fruits bitten by fruit bats, there is a high probability of becoming infected with NiV. According to the example of NiV passing through the body fluids and corpses of patients with Nipah disease [29], the farmers infected with NiV will become a new source of infection, making NiV spread rapidly from person to person and leading to an outbreak of Nipah disease in China.

## 4. Prospects for Research on Nipah Disease

China should pay attention to this emerging zoonosis. Although it was confirmed that ribavirin inhibits the replication of Nipah virus in vitro, it was found to have a therapeutic effect by a small number of patients and hamster model infection experiments [40]. While animal models are currently being used to simulate the pathogenesis and transmission mechanisms of NiV [63]. The high-risk susceptibility for transmission between humans is driven by specific human behaviors and the interactions between patients and caregivers. This is difficult to simulate in animal models. Therefore, animal models cannot be applied to forward propagation studies of humans [33]. Based on the NiV propagation pathway and the risk analysis of NiV in China, a propagation model that can be applied to human propagation research should be established in the future. In addition, according to research results published throughout the world, it is also necessary to develop and introduce an international standardized indirect ELISA kit and an IgM capturing ELISA to allow antibody detection of NiV to eliminate the risk of a Nipah disease outbreak in China.
